# Simplified Unified BARGE Method to Assess Migration of Phthalate Esters in Ingested PVC Consumer Products

**DOI:** 10.3390/ijerph20031907

**Published:** 2023-01-20

**Authors:** Dana Fahad M. S. Mohamed, Du Yung Kim, Jinsung An, Minhye Kim, Sa-Ho Chun, Jung-Hwan Kwon

**Affiliations:** 1 Division of Environmental Science and Ecological Engineering, Korea University, 145 Anam-ro, Seongbuk-gu, Seoul 02841, Republic of Korea; 2 Department of Civil and Environmental Engineering, Hanyang University, 55 Hanyangdeahak-ro, Sangnok-gu, Ansan 15588, Republic of Korea; 3 Chemical Products Team, FITI Testing and Research Institute, 21 Yangcheong 3-gil, Cheongju-si 28115, Republic of Korea

**Keywords:** bioaccessibility, migration, polyvinyl chloride, phthalate esters, unified BARGE method, microplastics, consumer exposure

## Abstract

The unified bioaccessibility research group of Europe (BARGE) method (UBM) suggests using in vitro experimental conditions for simulating the release of chemicals from confined matrices, such as soils and sediments, in the human gastrointestinal tract. It contains comprehensive steps that simulate human digestion pathways and has good potential for application in the leaching of plastic additives from accidentally ingested plastic particles. However, its complexity could be a challenge for routine screening assessments of the migration of chemicals from consumer plastic products. In this study, the UBM was modified to assess the migration of plastic additives from consumer products with five model phthalate esters (i.e., dibutyl phthalate (DBP), benzyl butyl phthalate (BBP), bis(2-ethylhexyl) phthalate (DEHP), and di-*n*-octyl phthalate (DNOP)) from polyvinyl chloride (PVC). The migration of phthalate esters was observed in four digestive phases (saliva, gastric, duodenal, and bile). Three separate experiments were conducted with the addition of (1) inorganic constituents only, (2) inorganic and organic constituents, and (3) inorganic and organic constituents in combination with digestive enzymes. While using enzymes with the UBM solution, the migrated mass for leached compounds was comparatively low (0.226 ± 0.04 μg) in most digestion phases, likely due to a self-generated coating of enzymes on the plastic materials. However, higher mass migration (0.301 ± 0.05) was observed when phthalate esters were analyzed in the UBM solution, excluding the enzymes. A ring test among six independent laboratories confirmed the robustness of the modified method. Therefore, we propose a simplified version of the original UBM designed mainly for the migration of inorganic elements using only the inorganic and organic components of the solution throughout all phases of digestion.

## 1. Introduction

The exposure of children to environmental contaminants is expected to differ from that of adults owing to their physiological and behavioral characteristics [1]. Infants and toddlers, specifically from 3 to 36 months of age, tend to engage in mouthing behaviors, which result in increased exposure to environmental contaminants [2,3]. The metabolic rates of children are faster than those of adults, which consequently leads to a faster breakdown. Infants and children are smaller than adults and thus receive a larger dose of these contaminants due to their food and water requirements per unit of body mass, hand-to-mouth activity, and ventilation rate [4,5]. Infants and toddlers tend to place various types of objects in their mouths such as plastic toys, pacifiers, and teethers. Plastic toys often contain a mixture of plasticizers, flame retardants, and antimicrobial substances [6,7,8]. Phthalate esters are a type of plasticizer that help improve the flexibility of plastic materials and some are classified as endocrine disrupting chemicals that cause developmental disorders [9]. They are not covalently bound to the polymer chain, allowing them to migrate from the product to the saliva when mouthed or to digestive fluids when ingested [6,10,11,12].

Many studies have focused on the total concentrations of contaminants in ingested matrices. However, this does not mean that all unbound chemicals in the product readily migrate and become bioaccessible in the gastrointestinal fluids. Not considering the migration of these unbound additives in the risk assessment of chemicals in consumer products can lead to a serious overestimation of exposure [13]. The bioaccessible fraction is defined as the maximum amount of contaminant that can be absorbed within the gastrointestinal tract [14], whereas the bioavailability means the proportion of the contaminant that enters the circulation when it is introduced into the body. Although relative bioavailability needs to be derived using animal models for site-specific human risk assessment of hazardous substances, economic/ethical issues have been continuously raised [15]. Thus, various in vitro test methods that can replace the in vivo toxicity test are proposed. As a representative in vitro method, the Bioaccessibility Research Group of Europe (BARGE) developed a unified method that simulates the human gastrointestinal tract under laboratory conditions. This method is used to determine the bioaccessibility of contaminants in soils in human health risk assessments in Europe [16].

The unified BARGE method (UBM) was developed primarily for evaluating the bioaccessibility of heavy metals accumulated in soil matrices [17,18]. To the best of our knowledge, few studies have used the UBM protocol to evaluate the migration of contaminants from orally ingested plastic consumer products. Owing to the increasing needs for risk assessment of chemicals in consumer products [19], it is necessary to extend the applicability of UBM. The UBM solution contains inorganic, organic, and enzymatic components in four digestion phases (saliva, gastric, duodenal, and bile fluids) that mimic the human digestive system and bring it to relatively simple in vitro systems. However, salts, soluble proteins, and enzymes may cause difficulties in extracting target analytes from test solutions, as has been observed in many earlier biomonitoring studies of hydrophobic organic compounds (HOCs) in tissue residue analysis [20,21,22]. Therefore, it is important to determine an optimized composition of digestive solutions for screening the bioaccessibility of HOCs from ingested products. Phthalate plasticizers, such as di(2-ethylhexyl) phthalate (DEHP), are known to cause adverse effects, even at low exposure concentrations [23,24]. As plasticizers are added in high percentages in plastic products, the careful experimental evaluation of bioaccessibility from accidentally ingested products is vital to estimate their exposure, especially for infants and toddlers.

Traditionally, sample preparation methods include liquid-liquid extraction (LLE) and solid-phase extraction (SPE) [25,26,27]. However, these methods do not agree with the current trends in green analytical chemistry [28], promoting the reduction in toxic and harmful solvents during the preparation and pre-treatment steps of analytical processes [29]. Other aspects (including those related to extraction efficiency, reproducibility, and cost) should be considered [30]. The main objectives of this study were (i) to evaluate which extraction method is most appropriate for the UBM, (ii) to obtain the extraction efficiency of the UBM protocol for the selected phthalate esters from polyvinyl chloride (PVC) consumer products by varying the solution compositions containing inorganic components only, both inorganic and organic components, or both inorganic and organic components with enzymes, and (iii) to compare the mass migrated out of PVC consumer products using experimental UBMs and those from mathematical exposure models. The robustness of the proposed modified UBM was evaluated using a ring test performed in six independent laboratories.

## 2. Materials and Methods

### 2.1. Chemicals and Materials

The US Environmental Protection Agency (EPA) 506 standard mixture of seven phthalates (benzyl butyl phthalate (BBP), di(2-ethylhexyl) (DEHP), dibutyl phthalate (DBP), diethyl phthalate (DEP), dimethyl phthalate (DMP), di-*n*-octyl phthalate (DnOP), and di(2-ethylhexyl) adipate (DEHA)) containing 1000 μg mL^−1^ of each chemical, dioctyl terephthalate (DOTP), and the internal standard (IS) (benzyl benzoate in 5000 μg mL^−1^ in *n*-hexane) was purchased from Fisher Scientific (Waltham, MA, USA). Methanol (≥99.9%, Sigma Aldrich, St. Louis, MO, USA) was used as a solvent for dilution, tetrahydrofuran (≥99.9%, Sigma Aldrich) was used to dissolve the PVC toys, and hexane (≥97.0%, Sigma Aldrich) was used as a solvent for phthalate extraction. The standards were diluted in 100% methanol to five concentrations (0.1, 1, 5, 10, and 50 μg mL^−1^) and the internal standard was prepared in hexane at 1 μg mL^−1^. During method development, working solutions were prepared by spiking standard solutions with ultrapure water or gastric (G) and gastrointestinal (GI) fluids.

The digestive fluids were prepared according to the UBM protocol. In the original UBM protocol, 250 mL of inorganic constituents and 250 mL of organic constituents are combined to produce a total volume of 500 mL. The solid enzymes were then added to the mixture of both constituents to mimic the digestion process. The experiment was conducted by adding inorganic (I), inorganic and organic (IO), and inorganic, organic, and enzyme (IOE) chemical solutions. For the analysis, I solution (5 mL) and deionized water (5 mL) were used. For the IO solution, inorganic (5 mL) and organic (5 mL) solvents were used. For the IOE solution, 10 mL of each of the combined solutions incubated with the enzymes were used. The reagents used for the composition of each digestive fluid are listed in Table 1.

PVC for phthalate analysis (CRM 113-03-006) was purchased from the Korea Research Institute of Standards and Science (Daejeon, Republic of Korea). The certified values in the reference material were 972, 962, 999, and 967 mg kg^−1^ for DBP, BBP, DEHP, and DNOP, respectively. The PVC material was ground to finer particles (208 ± 89 μm) using a freezer mill (6875 Freezer/Mill^®^, SPEX SamplePrep, LLC, Metuchen, NJ, USA) to establish the modified UBM. For external validation using the ring test, a sample of a consumer product containing 34.1% (*w*/*w*) DEHP was used.

### 2.2. Instrumental Analysis

The phthalate concentration was quantified using an Agilent 7890A gas chromatograph (GC) with a 5975C mass spectrometry detector (MSD) (Agilent Technologies, Santa Clara, CA, USA) for method validation. An Agilent DB-5MS capillary column (30 m × 0.25 mm; 0.35 μm film thickness) was used for separation. Helium was used as the carrier gas at 1 mL min^−1^. The inlet temperature was set at 300 °C and the pressure was 10.523 psi. The initial GC oven temperature was 100 °C and increased at a rate of 15 °C min^−1^ for 6.67 min till it reached 200 °C. It was then increased at a rate of 5 °C min^−1^ until it reached 270 °C. The total run time was 36.67 min per sample. The ions were monitored in scan and selective ion monitoring mode. The concentration of the phthalates were quantified using benzyl benzoate as an internal standard. The external standards were selected ranging from 0.1 μg mL^−1^ to 50 μg mL^−1^. The mass-to-charge ratios (*m*/*z*) were 149 and 223 for DBP, 149 and 206 for BBP, 149 and 167 for DEHP, 149 and 279 for DNOP, 149 and 167 for DOTP, and 212 and 213 for benzyl benzoate. The coefficients of determination for the calibration were greater than 0.99. All the samples were analyzed in triplicate. The analytes were not detected in the blank extractions of the digestive fluid. The absence of carry-over of analytes in GC-MS was evaluated using an injection of hexane as a control for each sample analysis. The detection limits were obtained as 0.14, 0.29, 0.09, 0.14, and 0.09 μg mL^−1^ for DBP, BBP, DEHP, DNOP, and DOTP, respectively. All the certified reference material and products tested contained sufficiently greater concentration of phthalates than their detection limits.

### 2.3. In Vitro Oral Bioaccessibility Test (UBM)

Following the UBM protocol (Bioaccesibility Research, 2011), artificial solutions mimicking saliva (S), gastric fluid (G), duodenal fluid (D), and bile (B) were prepared (detailed compositions are listed in Table 1). The components in each digestive fluid were dissolved in ultrapure water and completely mixed with a magnetic stirrer for 1 h. Each fluid was prepared separately to determine the maximum possible migration in each combination (i.e., inorganic constituents only, both inorganic and organic constituents, both inorganic and organic constituents with enzymes, etc.). To ensure the enzyme activity, the enzyme solutions were prepared freshly on the same day and incubated for 1 h at 37 ± 2 °C for activation before the start of the simulated digestion process. To analyze the extraction recovery, 1 mg L^−1^ of each phthalate was added to a 15 mL glass centrifuge tube. For the preparation of the gastric phase (pH 1.2 ± 0.1), 1.5 mL of saliva and 2.3 mL of gastric fluid were added and mixed in an end-over-end rotator for 1 h at 37 ± 2 °C. The intestinal phase (pH 6.3 ± 0.5) was prepared by adding 4.6 mL of duodenal fluid and 1.5 mL of bile fluid to the gastric phase and was mixed in an end-over-end rotator for 4 h. The pH of each phase was adjusted using 1 M NaOH and 37% HCl. The same procedure was used to analyze the mass migrated from 0.1 g of PVC reference material (CRM 113-03-006).

### 2.4. Analysis with Different Extraction Methods

#### 2.4.1. Liquid-Liquid Extraction at Different Volumes of Extraction Solvents

In accordance with green analytical chemistry, the solvents were tested at different volumes to determine whether the efficiency was appropriate even at smaller volumes.

##### Higher Volume of Extraction Solvent

A volume of 20 mL of spiked samples in the combined phases of digestion fluid was added to a separatory funnel and 10 mL of *n*-hexane was used to extract the analytes. This process was repeated 3 times and the combined supernatant was concentrated under a gentle nitrogen stream (1.0 mL) and subjected to GC/MS analysis.

##### Lower Volume of Extraction Solvent

The spiked samples (10 mL) were added to a 15 mL glass centrifuge tube and mixed with 1.0 mL *n*-hexane. The samples were vortexed for 2 min and centrifuged at 3000 rpm for 10 min. The supernatant was filtered through a 0.45 μm PTFE membrane filter (Advantec, Dublin, CA, USA) and the filtrate was subjected to GC/MS analysis.

### 2.5. Screening of the Migration of Phthalates in Children’s Toys Using the Simplified UBM

Four different bath toys were purchased online. The details of each item are presented in Table 2. The samples mainly contained an alternative phthalate plasticizer (DOTP). To measure the initial concentration of phthalates in the sample, 0.3 g of the sample was weighed and placed in a 40 mL glass vial. A volume of 10 mL of tetrahydrofuran (THF) was added and the solution was sonicated for 1 h. For the analyte extraction, 20 mL hexane was added to the THF solution and the mixture was left at room temperature for 30 min. The supernatant was filtered through a 0.45 μm PTFE syringe filter and subjected to GC/MS analysis.

After verifying the initial concentration of the toy samples, the mass that migrated during oral migration was analyzed similarly to the previous method using certified reference material. The samples were weighed up to 0.07 g and 1.5 mL of saliva and 2.3 mL of gastric fluid was added and mixed in an end-over-end rotator for 1 h at 37 ± 2 °C [16,31]. The intestinal phase (pH 6.3 ± 0.5) was prepared by adding 4.6 mL of duodenal fluid and 1.5 mL of bile to the gastric phase and was mixed in an end-over-end rotator for 4 h. The phthalates were extracted with 1 mL of *n*-hexane and the supernatant was subjected to GC/MS analysis.

### 2.6. Ring Test to Determine the Reproducibility of the Simplified UBM

Inter-laboratory validation of the method was conducted in six laboratories, including the Environmental Chemistry Lab where the modified UBM was developed. The ground-certified material was sent to each participating laboratory using the test protocol. The robustness of this method was evaluated using a robust Z-score.

robust Z-score = |(x − μ)/normalized IQR|(1)

normalized IQR = 0.743 × (3rd quartile value − 1st quartile value)(2)

where x is an individual amount of DEHP migrated from the certified material, μ is the median and standard deviation values of the six participating laboratories, and IQR is the interquartile range.

## 3. Results

### 3.1. Comparing Conventional Liquid-Liquid Extraction to Low Volume Liquid-Liquid Extraction

**Figure 1:**
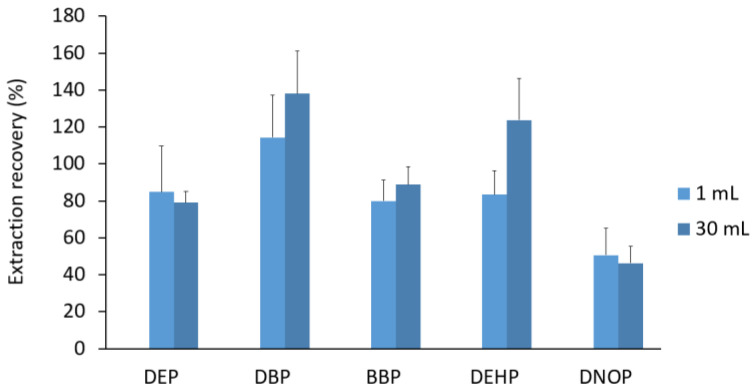
Comparison of extraction recovery using liquid-liquid extraction (LLE) with different volume of extracting solvent. Error bars represent standard deviations of triplicate analyses. DEP: diethyl phthalate, DBP: dibutyl phthalate, BBP: benzylbutyl phthalate, DEHP: di(2-ethylhexyl) phthalate, and DNOP: di(isononyl) phthalate.

The extractions at different volumes were compared to determine whether a simplified extraction method may be more appropriate for a standardized process. *n*-Hexane was used as an extractant at different volumes (1 and 30 mL) and the two methods were compared (Figure 1). Distilled water was spiked prior to the UBM protocol to determine the most efficient extraction method. The extraction recovery using 1 mL and 30 mL of solvent had similar results (both having an average extraction recovery of 85%). The lower volume was chosen as an ideal extraction method as it used a smaller volume of solvent and was performed in a shorter amount of time than conventional techniques. DNOP is a more hydrophobic analyte than the other phthalates, which could explain why the extraction efficiency was lower than that of the other analytes of interest (Figure 1). For both methods, the standard deviation was within the acceptable range. The relative standard deviation ranged 8–21% for 30 mL and 7–30% for 1 mL. Therefore, 1 mL of *n*-hexane was used for the extraction of phthalates in the UBM solution.

### 3.2. Comparison of Extraction Efficiency of Phthalates Extracted in Addition of Each UBM Solution

**Figure 2:**
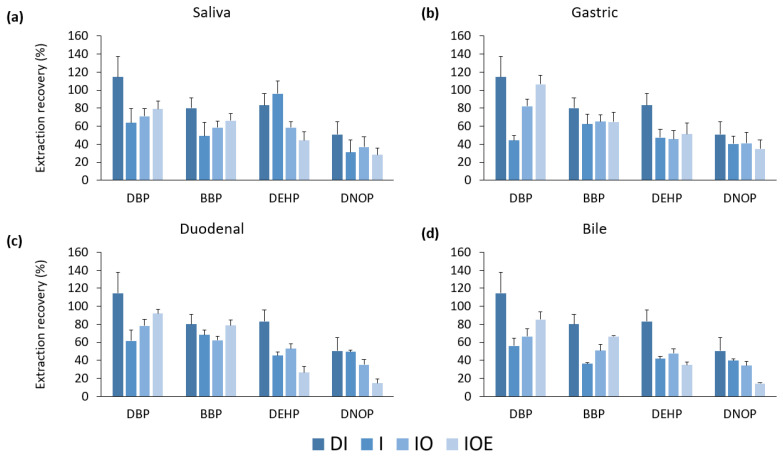
Extraction recovery with addition of each type of fluid (deionized water (DI), inorganic (I), inorganic and organic (IO), and inorganic, organic, and enzymes (IOE)) by each phase of digestion ((**a**) saliva, (**b**) gastric, (**c**) duodenal, and (**d**) bile fluids). Error bars represent standard deviations of triplicate analyses. DBP: dibutyl phthalate, BBP: benzylbutyl phthalate, DEHP: di(2-ethylhexyl) phthalate, and DNOP: di(isononyl) phthalate.

The UBM solution comprised various inorganic, organic, and enzymatic components. The phthalates were spiked, extracted, and analyzed by using four types of solutions: the first used deionized water, second only used the inorganic solution, third used the inorganic solution with the addition of the organic solution, and lastly used the original method, which included all of the inorganic, organic, and enzymatic solutions. Figure 2 compares the extraction efficiencies obtained for the four different solutions.

Although there were scatters, the extraction recovery in deionized water was generally the greatest. As more components were added to the UBM solution, the recovery decreased. Another trend was found to be lower for the more hydrophobic phthalates (DEHP and DNOP) than for the less hydrophobic phthalates (DBP and BBP). In the solution containing enzymes, the extraction recovery was the lowest; this was more apparent for the more hydrophobic DEHP and DNOP. This is likely because these hydrophobic analytes were not extracted by the solvent, as they may bind to proteins and other polymeric substances in the matrix [31].

### 3.3. Measuring the Mass Migration Using PVC-Certified Reference Material

**Figure 3:**
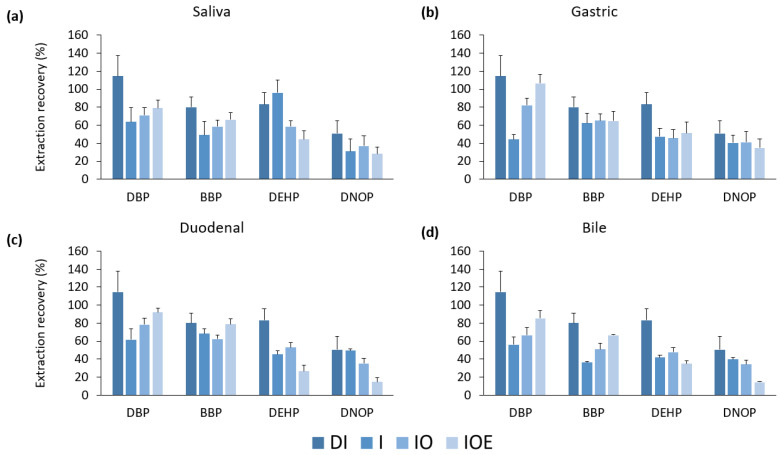
Mass of phthalates leached from PVC-certified reference material with addition of each fluid (deionized water, inorganic, inorganic and organic, and inorganic, organic, and enzymes) by each phase of digestion ((**a**) saliva, (**b**) gastric, (**c**) duodenal, and (**d**) bile fluids). Error bars represent standard deviations of triplicate analyses. DBP: dibutyl phthalate, BBP: benzylbutyl phthalate, DEHP: di(2-ethylhexyl) phthalate, and DNOP: di(isononyl) phthalate.

In contrast to the previous results, the results varied in the presence of a solid plastic material. Excluding the saliva solution, the mass migration was lower in the solution containing enzymes than in the inorganic and organic (IO) solution when 0.1 g of the PVC-certified material was added to the matrix. In the duodenal and bile fluid, the mass that migrated to the BBP and DNOP was lower (Figure 3).

**Figure 4:**
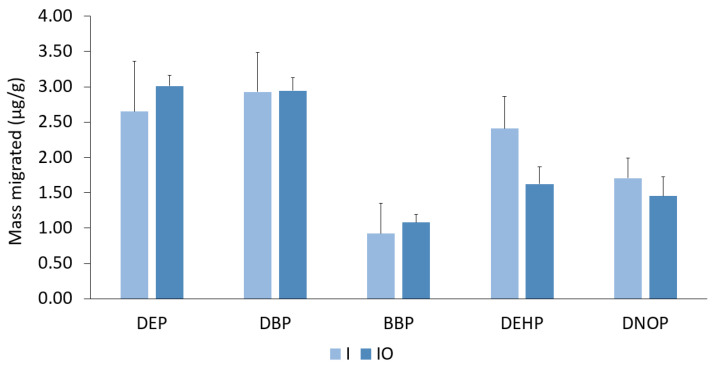
Sequential extraction of the reference material using inorganic (I) and inorganic and organic (IO) digestive fluid. Error bars represent standard deviation of triplicate analyses.

Upon observation of this solution, the enzymes formed a coat around the reference material that may have inhibited the leaching of phthalates. Since the UBM protocol was originally designed to determine the bioavailability of inorganic elements such as heavy metals, it may not necessarily be applicable when observing the maximum possible migration of phthalate esters from consumer products. Sequential extraction following the addition of each fluid was conducted by comparing inorganic fluid (I) with inorganic and organic fluid (IO) (Figure 4). The mass migrated was similar between the two solutions; therefore, a solution containing inorganic and organic materials can be used to analyze the migration of phthalates from consumer products. Approximately 0.2% of phthalates were leached from the certified reference material into the artificial gastrointestinal matrix.

### 3.4. Validation of the Modified UBM Using a Ring Test

Each participating laboratory conducted triplicate analyses using the same reference materials. The robust Z-scores were 0.33, 0.33, 0.65, 0.93, 1.26, and 5.12. As it is regarded that the performance of the experimental results is satisfactory when the robust Z-score is less than 2.0 [32], the proposed method is likely to be conducted providing robust results. One participating laboratory reported a very high migrated mass compared with the other five laboratories, which might be due to the grinding step of the reference material.

### 3.5. Using the Simplified Unified BARGE Method for the Screening of the Migration of Phthalate Esters from Children’s Toys

Four toys were tested using inorganic and organic matrices. Sample 1 had a lower concentration of DOTP present in the sample in comparison to the other samples. Therefore, the migrated mass of DOTP was 2.02 ± 0.57 μg. The other samples had concentrations of 926 ± 296, 1223 ± 530, and 907 ± 181 μg for samples 2, 3, and 4, respectively. Approximately 3–5% (*w*/*w*) of the DOTP migrated into the artificial gastrointestinal fluid during the extraction period.

### 3.6. Estimating Mouthing Exposure to Phthalates in Children’s Toys

**Figure 5:**
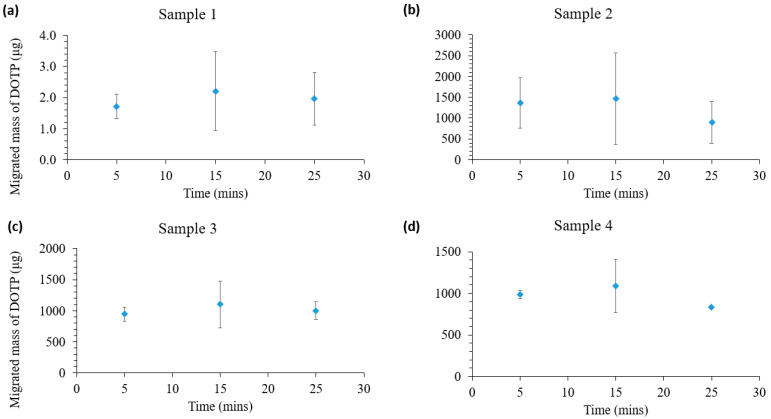
Migrated mass of DOTP in saliva at different durations. Error bars represent standard deviations of triplicate analyses.

The results of the phthalate ester migration from the toy products into saliva are shown in Figure 5. The samples listed in Table 1 were used in this process. The amount of DOTP that migrated immediately within 5 min of exposure and the migrated mass of DOTP between 5 and 25 min were not statistically significant. The average masses of DOTP between 5 and 25 min were 1.963± 0.246, 1244.7 ± 302.91, 1017.83 ± 78.3, and 968.3 ± 130.49 μg for samples 1, 2, 3, and 4, respectively. These results demonstrate that the migrated mass of DOTP was not significantly affected by the experimental time.

## 4. Discussion

Many studies have been conducted on children’s exposure to PVC mouthing articles [12,33,34]. However, there are few studies involving the in vitro digestion of children’s products containing phthalates [35]. In this study, steps were followed according to the UBM digestion protocol to mimic human digestion pathways. Following green analytical chemistry, the use of a lower volume of solvent has been found to be sufficient to attain appropriate extraction recovery [29]. Although studies have been conducted using the UBM to measure heavy metals in soil matrices [17,18], this study mainly focused on the oral exposure of consumer products and their debris with experimental reproducibility. The digested samples were extracted using a lower volume of solvent (1 mL) after the extraction efficiency was compared using 1 and 30 mL of the solvent. The oral exposure presented was built upon a previous work [31] with a similar method using a lower volume of extraction solvent for phthalates in the UBM solution. However, this study included an additional step of analyzing the mass migrated from the PVC products. As plastic matrices can be quite complex, the conventional UBM was modified to simulate the maximum migration of leached phthalates to the body. This was broken down by each component of the digestive fluid and inorganic, organic, and enzymatic components of the liquid. Although it is possible to obtain a high extraction efficiency of spiked phthalates in the UBM matrix, it is difficult to determine the leached phthalates in the case of a solid sample. The UBM protocol using inorganic and organic (IO) solutions can be used as a standardized method to determine the maximum amount leached from consumer products. This modified method may be used in the future for other types of hydrophobic endocrine disruptors (EDCs) such as organophosphate flame retardants and various UV stabilizers.

As observed during the ring test, the size and shape of the ground particles are important in determining the total mass of migrated DEHP during in vitro digestion. As it is well acknowledged that the leaching of plastic additives and associated contaminants from microplastics is strongly influenced by particle sizes [36,37,38], further research is necessary to identify the size range of microplastics accidentally ingested via mouthing behavior in children.

In compliance with the ban on phthalates, many toys were found to use DOTP as an alternative to phthalate (DEHP) [39,40]. Therefore, the mass migration of DOTP from PVC according to children’s mouthing behavior was also observed. The migrations at different times were compared to determine whether the mouthing duration may affect the migration of DOTP from PVC. The saliva phase from the original UBM method was used and the migrated mass did not differ at different mouthing durations. Therefore, DOTP migration occurs immediately and does not increase with a longer mouthing duration.

## 5. Conclusions

Low volume liquid-liquid extraction was used as it is more environmentally friendly, time effective, and has an easier sample clean up. The UBM solution spiked with phthalate had the highest extraction recovery in the enzyme-containing solution but does not apply to consumer products. Applying this method to a solid PVC reference material resulted in the formation of a coating on the material that inhibited the leaching of phthalate esters, causing a lower migrated mass. After analysis, we found that excluding the enzymatic components resulted in clearer and more consistent results in all the phases of digestion and the modified UBM was found to be robust in a ring test in six different laboratories. The UBM protocol using inorganic and organic solutions can be used as a more reproducible method to determine the maximum quantity leached from consumer products.

## Figures and Tables

**Table 1:** Composition of digestive fluids (S, G, D, and B) in 250 mL inorganic solution (I) and 250 mL of organic solution (O).

	Reagent	Saliva (S)	Gastric (G)	Duodenal (D)	Bile (B)
Inorganic constituents (250 mL)	KCl	448 mg	412 mg	282 mg	188 mg
	NaH_2_PO_4_	444 mg	133 mg		
	KSCN	100 mg			
	Na_2_SO_4_	285 mg			
	NaCl	149 mg	1376 mg	3506 mg	2630 mg
	CaCl_2_		200 mg		
	NH_4_Cl		153 mg		
	NaHCO_3_			2803.5 mg	2893 mg
	KH_2_PO_4_			40 mg	
	MgCl_2_			25 mg	
	NaOH (1 M)	0.9 mL			
	HCl (37%)		4.15 mL	90 μL	90 μL
Organic constituents (250 mL)	Urea	100 mg	42.5 mg	50 mg	125 mg
	Glucose		325 mg		
	Glucuronic acid		10 mg		
	Glucosamine hydrochloride		165 mg		
Enzymes	α-amylase	72.5 mg			
	Mucin	25 mg	1500 mg		
	Uric acid	7.5 mg			
	Bovine serum albumin		500 mg	500 mg	900 mg
	Pepsin		500 mg		
	CaCl_2_			100 mg	111 mg
	Pancreatin			1500 mg	
	Lipase			250 mg	
	Bile salts (Bovine)				3000 mg
pH ^1^		6.5 ± 0.5	1.1 ± 0.1	7.4 ± 0.2	8.0 ± 0.2

^1^ values after mixing inorganic and organic solutions.

**Table 2:** Description of PVC bath toy samples used for this experiment with the dioctyl terephthalate (DOTP) concentration.

Toy Sample ID	Description	Concentration of DOTP (μg)
1	Bath toy playset 1	50 ± 10
2	Bath toy playset 2	26,600 ± 1500
3	Rubber duck 1	23,100 ± 2960
4	Rubber duck 2	22,500 ± 500

## Data Availability

Data are available from authors upon request.
